# Twenty-six circulating antigens and two novel diagnostic candidate molecules identified in the serum of canines with experimental acute toxoplasmosis

**DOI:** 10.1186/s13071-016-1643-x

**Published:** 2016-06-29

**Authors:** Junxin Xue, Wei Jiang, Yongjun Chen, Yingchun Liu, Huajing Zhang, Yan Xiao, Yuanbiao Qiao, Kehe Huang, Quan Wang

**Affiliations:** College of Veterinary Medicine, Nanjing Agricultural University, Nanjing, 210095 China; Shanghai Veterinary Research Institute, Chinese Academy of Agricultural Science, Shanghai, 200241 China; College of Food Sciences, Shanghai University, Shanghai, 200444 China

**Keywords:** *Toxoplasma gondii*, Acute infection, Proteomics, Circulating antigens, Diagnostic candidates

## Abstract

**Background:**

The protozoan *Toxoplasma gondii* is a pathogen that causes severe opportunistic disease in a wide range of hosts. Efficient methods to diagnose acute *T. gondii* infection are essential for the administration of appropriate treatments and to reduce economic losses. In animals with acute infections, circulating antigens (CAgs) were detected as early as two days post-infection; these CAgs were reliable diagnostic indicators of acute infection. However, only a limited number of CAgs have been identified to date. The objective of this study was to identify a broader spectrum of CAgs and to explore novel diagnostic candidates in serum.

**Methods:**

A canine model of acute toxoplasmiosis was established. For this purpose, six dogs were infected by intraperitoneal inoculation of tachyzoites. The CAgs spectrum in the serum was identified with the immunoprecipitation-shotgun approach. Two CAgs with low homology to other species, coronin protein (TgCOR) and ELMO protein (TgELMO), were heterologously expressed in *Escherichia coli*. Polyclonal antibodies against these two proteins were prepared, and the presence of these proteins in the serum was verified by Western blotting. The two CAgs were detected and evaluated by indirect ELISA methods.

**Results:**

The CAgs levels peaked between two and five days after inoculation, and twenty-six CAgs were identified. Western blotting showed the presence of the two proteins in the serum during acute infection. Based on ELISA tests, the two CAgs were detected during acute infection.

**Conclusions:**

We identified twenty-six CAgs in the serum of canines with experimental acute toxoplasmosis and discovered two novel diagnostic candidates. We also provide new insights into the diagnosis of acute toxoplasmosis.

**Electronic supplementary material:**

The online version of this article (doi:10.1186/s13071-016-1643-x) contains supplementary material, which is available to authorized users.

## Background

*Toxoplasma gondii* can cause serious opportunistic infections in a wide range of hosts [1]. This species has tremendous disease-causing potential because it can invade any nucleated cell, resulting in lysis of the host cells [1]. The symptoms of *T. gondii* acute infection in humans range from mild flu-like symptoms in most people to more severe complications in immunocompromised individuals or following trans-placental transmission to a foetus [2, 3]. During the first or second trimester of pregnancy, infection with *T. gondii* could potentially lead to the parasite passing through the placenta to the foetus, resulting in mental retardation, retinochoroiditis, blindness, and even death [4, 5]. The available therapies of acute toxoplasmosis are not fully effective. Thus, it is crucial to diagnose *T. gondii* infection and make efforts to reduce toxoplasmosis transmission [6]. If an acute infection cannot be diagnosed in time, the optimal period for treatment will also be missed [7].

Laboratory tests are the main diagnostic method for acute *T. gondii* infection. Indirect (serological methods) and direct (PCR, *in situ* hybridization, isolation and histology) methods have been used to diagnose acute *T. gondii* infection [8]. In general, serological diagnosis methods are based on the detection of specific antibodies (IgG, IgM and IgA) [9, 10]. Antibodies are commonly produced in the late stage of acute infection or during chronic infection. Detection based on molecular biology methods is limited because specific equipment is required that is not universally available in clinical laboratories. Isolation and identification of the parasite requires extensive experience and is time-consuming. The lack of efficient diagnostic methods for acute *T. gondii* infection can lead to therapy failure, and there is an urgent need to identify new diagnostic candidates.

The *T. gondii* invasion process is rapid and dynamic and relies on the secretion of numerous proteins from micronemes, rhoptries and dense granules [11]. Excretory/secretory antigens (ESA) are a group of antigens from *T. gondii* that represent the majority of circulating antigens (CAgs) in the sera of hosts during acute toxoplasmosis and the reactivation of infection [12–14]. Recent evidence strongly suggests that CAgs can serve as serological markers to diagnose acute infection [15, 16]. To date, only a limited number of CAgs have been identified. Moreover, the identification of CAgs is meaningful for the diagnosis of acute *T. gondii* infections. The aim of this study was to identify the spectrum of *T. gondii* CAgs using the immunoprecipitation-LC-MS/MS technique. We also evaluated the use of antibodies to identify novel candidate molecules for the detection of acute toxoplasmosis by ELISA tests.

## Methods

### *T. gondii*, cell culture and animal preparations

The *T. gondii* RH strain is an international standard virulent strain that is widely used for *Toxoplasma* analysis. A virulent strain is also advantageous to successfully establish an acute *T. gondii* infection model. The *T. gondii* strain RH and Vero cells were stored in our laboratory. Vero cells were propagated at 37 °C in a 5 % CO_2_ atmosphere in DMEM (Dulbecco modified Eagle medium) supplemented with 10 % FBS, 2 mmol/l glutamine, 100 kU/l streptomycin, and 400 kU/l penicillin. *T. gondii* were grown and maintained in Vero cells.

Female BALB/c mice (~25 g body weight) were purchased from the Shanghai Laboratory Animal Centre at the Chinese Academy of Science (Shanghai, China). Six clean female beagles (3 months old; ~4 kg body weight) without pathogens that obviously interfere with scientific experiments were purchased from the Shanghai Xingang Laboratory Animal Farm (Shanghai, China). All of the animals used in the experiments were raised in a sterilized room and fed sterilized food and water at the Animal Laboratory Centre at the Shanghai Veterinary Research Institute. The study was approved by the Animal Care and Use Committee of the Shanghai Veterinary Research Institute. The animals were handled in strict accordance with the animal protection law of the People’s Republic of China (released on 09/18/2009) and the National Standards for Laboratory Animals in China (executed on 05/1/2002).

### Excretory/secretory antigens and antisera preparation

Excretory/secretory antigens were prepared according to previously described methods [14]. Briefly, 3 × 10^9^ purified tachyzoites were suspended in 15 ml DMEM and incubated at 37 °C for 2 h. Tachyzoites were removed by centrifugation at 1,000 g for 15 min (at 4 °C). The supernatant was supplemented with a protease inhibitor cocktail (Merck, Darmstadt, Germany, Cat No. 535140) and concentrated to 500 μl using a 3 kDa centrifugal filter (Merck, Cat No. UFC900308). The ESA protein concentration was determined using a DC protein assay reagent package (Bio-Rad, Hercules, USA, Cat No. 500-0120). Two groups of two female BALB/c mice were immunized. Two mice were immunised with EAS and two mice were immunised with PBS as a control. The 206 adjuvant (Seppic, Paris, France) was used according to the manufacturer’s instructions. In total, 100 μg ESA was injected into each mouse at 0, 2, 4, 6 and 8 weeks. Equal doses of PBS were used to immunize the control group mice. Blood sera were sampled from the tails of mice at 10 days after each immunization. To detect the ESA antisera, immunoblotting and ELISA assays were performed using standard methods.

### Development of acute infection models

Three dogs that were intraperitoneally inoculated with 1 × 10^9^ purified tachyzoites were treated as the test group. Three dogs that were intraperitoneally inoculated with the same volume of sterile PBS were treated as a control group. A 3 ml volume of blood was collected twice per day between days 1 and 3 and once per day between days 4 and 18. Blood was then collected once every 3 days until two months had elapsed. Each blood sample was divided into 2 tubes (one tube for serum separation and one tube for sodium citrate anticoagulation). The sera were used to detect CAgs or to purify CAgs by immunoprecipitation. Centrifuged sera (4,000 rpm for 10 min) from blood not treated with an anticoagulant were supplemented with a protease inhibitor cocktail (Merck, Cat No. 535140) and stored at -80 °C until use. The anticoagulant blood was used to extract DNA for *T. gondii* detection.

### Evaluation of an acute infection model by ELISA and nested PCR

To test for CAgs, an ELISA assay was performed using a commercial Circulating Antigen Detection ELISA (CA-ELISA) kit (Combined Company, Shenzhen, China). The results are presented as the mean value and standard error (SE) of the sample absorbance from 3 dog groups.

To detect *T. gondii* in the blood, a genomic DNA extraction kit (SBS Gentech, Shanghai, China) was used to extract DNA from whole blood samples. Nested-PCR was conducted as described in our earlier study [17]. The protozoa rDNA sequence contains 5'-IGS-18S rDNA-ITS1-5.8S rDNA-ITS2-28S rDNA-IGS-3'. Based on the sequence of the ITS1-5.8S rDNA-ITS2 gene (GenBank Accession No. X75453) of *T. gondii*, two pairs of nested PCR primers were designed. The forward primer 5'-ACC TTT GAA TCC CAA GCA-3' and the reverse primer 5'-TAA ATC GGA CAA ACG CCC-3' were used to amplify an 855 bp fragment representing the outer amplicon. The forward primer 5'-TTT GCA TTC AAG AAG CGT G-3' and the reverse primer 5'-AAG GTG CCA TTT GCG TTC-3' were used to amplify a 432 bp fragment representing the inner amplicon. The primers were used at 0.4 μM each in the 25 μl reaction system. Each reaction used 1 μl of the extracted DNA, 9.5 μl double-distilled water, 12.5 μl of the 2 × PCR mix (Dongsheng Biotech, Guangzhou, China), which consisted of 100 mM KCl, 20 mM Tris-HCl, 3 mM MgCl_2_, 400 μM dNTPs and 0.1U/μl Taq DNA polymerase. After an initial denaturation at 94 °C for 5 min, amplification consisted of 30 cycles of denaturation at 94 °C for 50 s, annealing at 55 °C for 40 s and extension at 72 °C for 45 s. Finally, the DNA fragments were extended at 72 °C for 10 min.

### Immunoprecipitation

Serum (10 μl) was sampled on days 2 and 3 post-challenge with *T. gondii*. Sera from each of the 3 beagles were combined (60 μl). The mixed serum was diluted with 540 μl sterile PBS (pH 7.4). Protein A agarose beads (100 μl, 25 %, Beyotime, Nantong, China, Cat No. P2006) were added, and the sample was stirred at 4 °C for 2 h to eliminate serum antibodies. The mixture was centrifuged at 4 °C at 2,500 rpm for 5 min. After that, the supernatant was collected. Protein G agarose beads (100 μl, 25 %, Beyotime, Cat No. P2009) and 5 μl (1 μg) normal mouse IgG was added to eliminate non-specific binding and then the sample was agitated at 4 °C for 2 h. After centrifugation, the supernatant was collected as described above. Then, 5 μl ESA antiserum was added to the supernatant and the mixture was stirred at 4 °C overnight. Protein G agarose beads (100 μl, 25 %, Beyotime) were added and then stirred at 4 °C for 3 h. The mixture was centrifuged at 2,500 rpm for 5 min to collect the beads. The beads were washed 3 times with PBS and then boiled with 60 μl sodium dodecylsulfate polyacrylamide gel electrophoresis (SDS-PAGE) sample buffer for 5 min. The sample was centrifuged at 2,500 rpm for 5 min, and the supernatant was collected for LC-MS/MS detection and 8 % polyacrylamide gel electrophoresis analysis.

### Filter-aided proteome preparation

The supernatant was supplemented with DTT (to a final concentration of 100 mM) and boiled for 5 min. After cooling, 200 μl UA buffer (8 M Urea, 150 mM Tris-HCl, pH 8.0) was added. The sample was then mixed. The liquid was transferred to a 10 kDa Millipore Amicon Ultra-4 centrifugal filter device and centrifuged at 14,000 rpm for 15 min. Another 200 μl UA buffer were added, and the sample was centrifuged as described above. The filtrate was discarded, and 100 μl iodoacetamide (50 mM iodoacetamide in UA) was added. The tube was shaken at 600 rpm for 1 min and then incubated at room temperature in the dark for 30 min. After being centrifuged at 14,000 rpm for 10 min, the solution was dialyzed against 100 μl UA buffer twice and then dialyzed against 100 μl dissolution buffer (25 mM NH_4_HCO_3_) 3 times. The mixture treated with 40 μl buffered trypsin (2 μg trypsin in 40 μl dissolution buffer) and then incubated at 37 °C for 18 h. The incubated solution was centrifuged at 14,000 rpm for 10 min, and the supernatant was used for LC-MS/MS analysis [18].

### Liquid chromatography (LC) - electrospray ionization (ESI) tandem MS (MS/MS) analysis using a Q Exactive mass spectrometer

Experiments were performed using a Q Exactive mass spectrometer coupled to an Easy nLC (Thermo Fisher Scientific, Waltham, USA). The peptide mixture was loaded onto a C18-reverse phase column (15 cm long, 75 μg inner diameter) packed in-house with RP-C18 5 μm resin in buffer A (0.1 % formic acid in HPLC-grade water). Peptides were separated with a linear gradient of buffer B (0.1 % Formic acid in 84 % acetonitrile) at a flow rate of 250 nl/min over 60 min. MS data were acquired using a data-dependent top10 method by dynamically choosing the most abundant precursor ions from the survey scan (300–1800 m/z) for HCD (Higher-energy collisional dissociation) fragmentation. Determination of the target value was based on predictive Automatic Gain Control (pAGC). The dynamic exclusion duration was 20 s. The survey scans were acquired at a resolution of 70,000 at 200 m/z. The resolution for the HCD spectra was set to 17,500 at 200 m/z. The normalized collision energy was 27 eV. The underfill ratio, which specifies the minimum percentage of the target value likely to be reached at the maximum fill time, was defined as 0.1 %. The instrument was run with the peptide recognition mode enabled.

### ESI mass spectrometry data analysis

The MS/MS spectra were used to search the UniProt database (26,517 sequences, downloaded on June 13th, 2014) with the MASCOT engine (Matrix Science, version 2.2). To identify proteins, the following options were used: Peptide mass tolerance = 20 ppm; MS/MS tolerance = 0.1 Da; Enzyme = Trypsin; Missed cleavage = 2; Fixed modification: carbamidomethyl (C); Variable modification: Oxidation (M); mascot score ≥ 20; and FDR < 0.01 at the peptide and protein levels.

### Bioinformatics analysis

To better understand the biological functions of the identified proteins, GO annotation was performed based on BLAST results using the ToxoDB (http://toxodb.org/toxo/), QuickGO (http://www.ebi.ac.uk/QuickGO) and DAVID v. 6.7 (http://david.abcc.ncifcrf.gov) databases [19–21].

### Protein expression and antibody production

To further verify the CAgs components present in the serum, coronin protein (TgCOR) and ELMO/CED 12 family protein (TgELMO), which both have low homology to other species were selected for further evaluation of their diagnostic value. The secondary structures of TgCOR (GenBank Accession No. EPT25607.1) and TgELMO (GenBank Accession No. XP_002367438.1) were analysed using Protean DNAStar software. Hydrophilicity, surface accessibility and antigenicity prediction schemes were used. TgCOR and TgELMO were expressed using an *E. coli* (BL 21) expression system and the pET-28a-c(+) vector. The recombinant plasmids were sent to Invitrogen Company for full-length sequencing. The protein-coding regions, primers, restriction enzymes and cut sites, expression data and the theoretical molecular weights of the proteins are shown in Table 1. The transformed cells with expression vectors were grown in LB medium containing 50 μg/ml ampicillin at 37 °C with vigorous shaking until the optical density (600 nm) of the medium reached 0.6. The expression was induced by 0.5 mM isopropyl β-D-1-thiogalactopyranoside (IPTG) and the culture was incubated at 20 °C for 18 h. Collected cells were lysed by sonication. Recombinant proteins isolated from inclusion bodies were refolded by a refolding kit (Novagen, Darmstadt, Germany, CAT No. 70123-3) and purified using Ni-NTA His-Bind resin (Novagen). Recombinant proteins were dialysed and endotoxin was removed using the Triton-X114 method [22]. Briefly, Triton X-114 was added to the protein solution (final concentration of Triton X 114 was 2 %, W/V). After being mixed by vortex, the solution was incubated on ice for 15 min and then at 50 °C for 5 min. The supernatant was collected after centrifugation (12,000 *g*, 1 min, 30 °C. Protein concentrations were measured using a DC protein assay reagent kit (Bio-Rad, USA). BALB/c mice were immunized with each protein at a dose of 100 μg per mouse. The immunization procedures and the detection of antibody titres (Table 1) were performed as described above. Blood samples were collected 10 days after the second booster injection.

**Table 1:** Information of expressed protein

Protein name	Expressed region	Primers^a^	Restriction enzyme	Form of expression	Recombinant protein weight (Da)	Antiserum dillution
TgCOR	H211-A621	FP:55'-GTCGGATCCCACGATGGATCGAAGGCAT -3'	*BamHI*	Inclusion body	50,494.01	1:8000
		RP: 5'-CGCCTCGAGAGCGGCTTCGTCCTGA -3'	*XhoI*			
TgELMO	G1613 -L1916	FP:5'- TCTGGATCCGGGTCTCCGAGGTA -3'	*BamHI*	Soluble	39,203.52	1:4000
		RP: 5'- AAGGTCGACTAGACCGGACGGAG -3'	*SalI*			

^a^Enzyme cleavage sites were underlined

### Verification of circulating antigens in the sera in an acute infection model

To verify the presence of the identified CAgs in the sera of animals with acute *T. gondii* infections, sera from the test group were analysed by Western blotting at a dilution of 1:10. Mixed serum from the control group was used as a negative control. Antiserum for TgCOR and TgELMO was treated as the primary antibody at a dilution of 1:2,000 and 1:4,000, respectively. HRP-conjugated goat anti-mouse IgG (Jackson, USA) was used as a secondary antibody at a dilution of 1:4,000. The detection of signals on the membranes was performed using an Enhanced Chemiluminescence (ECL) Kit (Thermo Fisher Scientific, Waltham, USA).

### Analysis of the diagnostic value of TgCOR and TgELMO proteins

To evaluate the use of ELISA methods for clinical diagnosis, sera collected from the 6 dogs in the initial 16 days post-infection were assessed for CAgs. Microwell plates with 96 wells were coated (100 μl/well) with serum samples in carbonate buffer at a dilution of 1:50. TgCOR and TgELMO antiserum was used as the primary antibody at a dilution of 1:1,000 and 1:2,000, respectively. HRP-conjugated goat anti-mouse IgG (Jackson, USA) was used as a secondary antibody at a dilution of 1:4,000. The results are presented as the absorbance value for each sample.

## Results

### An acute model of *T. gondii* infection in canines using inoculation with tachyzoites was established successfully

**Fig. 1:**
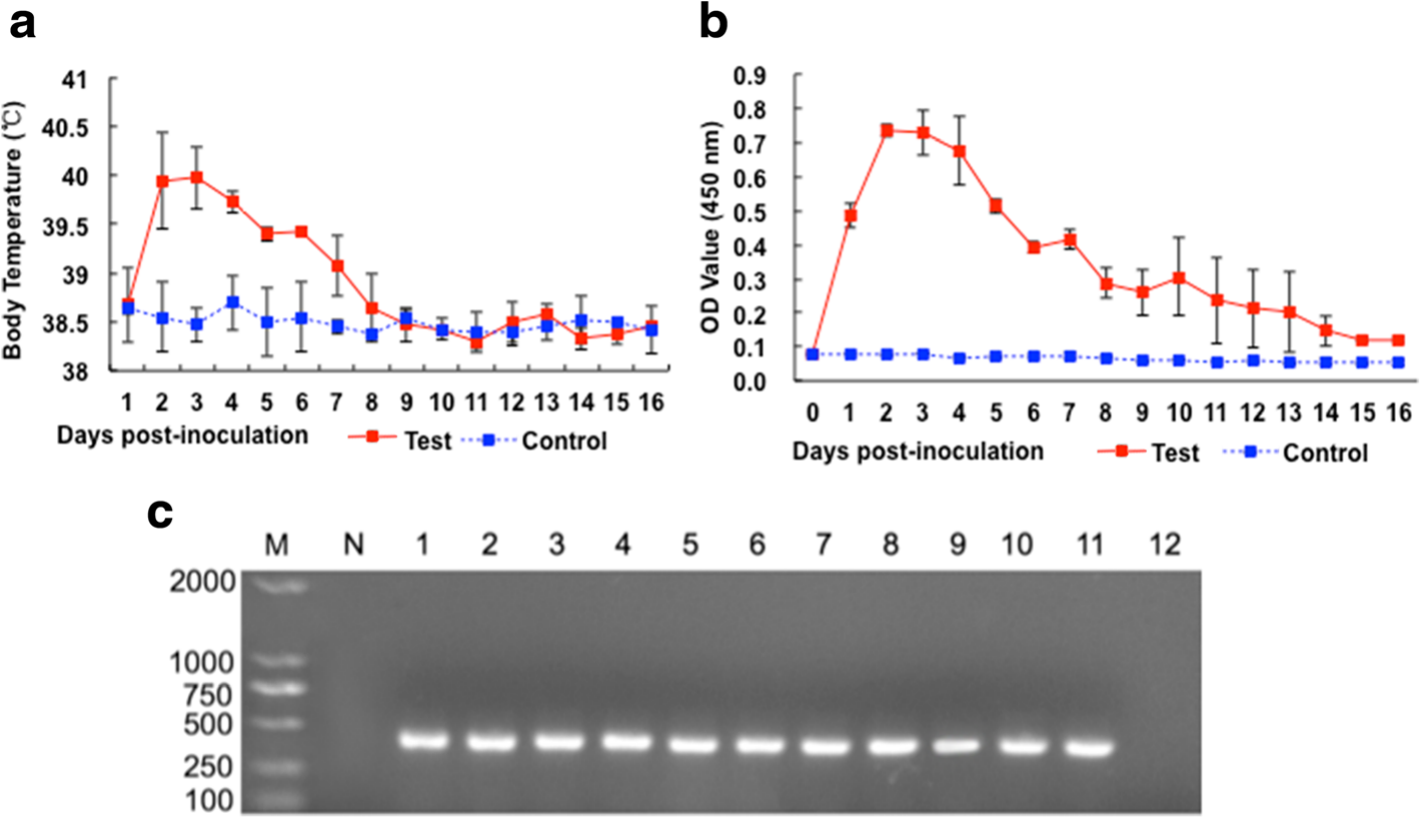
Analyses of body temperature, CAgs and nested-PCR. **a** Body temperature between 1 to 16 days post-infection. **b** Sera from days 0 to 16 were tested for CAgs levels using a commercial Circulating Antigen Detection ELISA (CA-ELISA) Kit. Values represent the mean and standard error from two groups. **c** Nested-PCR results. Lane M: marker; Lane N: negative control; Lanes 1–12: Nested-PCR results from day 1 to day 12 post-inoculation. The nested-PCR results from one dog in the test group are shown

The *T. gondii* strain RH is lethal when used to inoculate mice; the death rate is also high in rabbit models of acute RH strain infection. Small rabbits are also unsuitable for the continuous collection of large amounts of blood. To ensure the collection of adequate amounts of sera, an acute canine infection model was established in this trial. Our early experiments showed that dogs (~7 kg body weight) did not display symptoms of acute infection if inoculated with less than 1 × 10^8^ tachyzoites. In this study, the dogs showed fever symptoms 24 h post-*T. gondii* infection (Fig. 1a). *Toxoplasma gondii* and CAgs could be detected in the blood after infection. The levels of CAgs peaked between 2 and 5 days after inoculation. The curve of CAgs levels was similar to that of body temperature. CAgs were gradually cleared away concomitant with the disappearance of *T. gondii* (Fig. 1b, c). These results demonstrate the successful development of an acute *T. gondii* infection model.

### Circulating antigens enriched and purified by immunoprecipitation were identified by LC-MS/MS

**Fig. 2:**
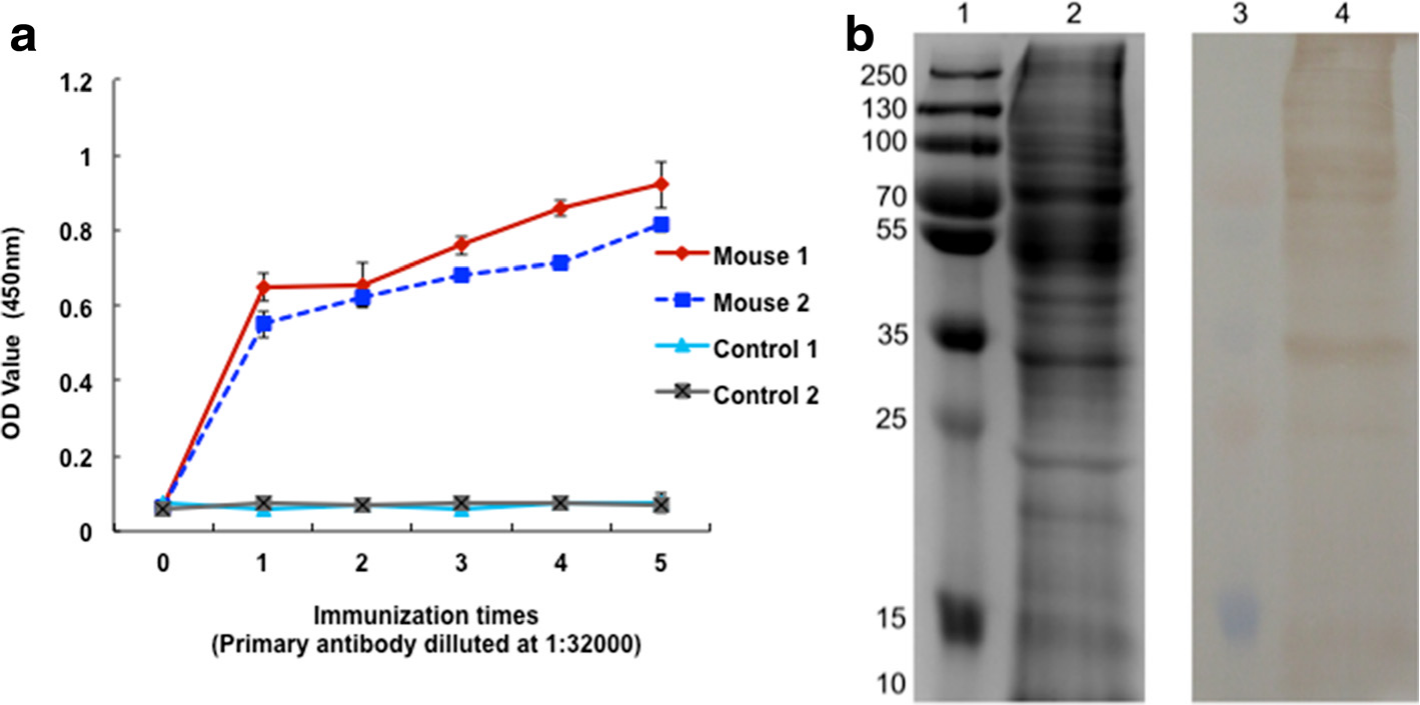
ESA antibodies were tested by ELISA and immunoblotting analyses. **a** Levels of ESA antibodies after each immunization. Two groups of 2 female BALB/c mice were immunized five times at 2-week intervals. PBS was used to immunize 2 mice in the control group. Blood sera were sampled from the tails of mice 10 days after immunization. Each sample was measured in duplicate. Values represent the mean and standard deviation. **b** Western blotting analysis of ESA. Lane 1: marker on gel; Lane 2: ESA on gel; Lane 3: marker on NC membrane; Lane 4: ESA on NC membrane. The membranes were coloured with 3, 3’-diaminobenzidine

**Fig. 3:**
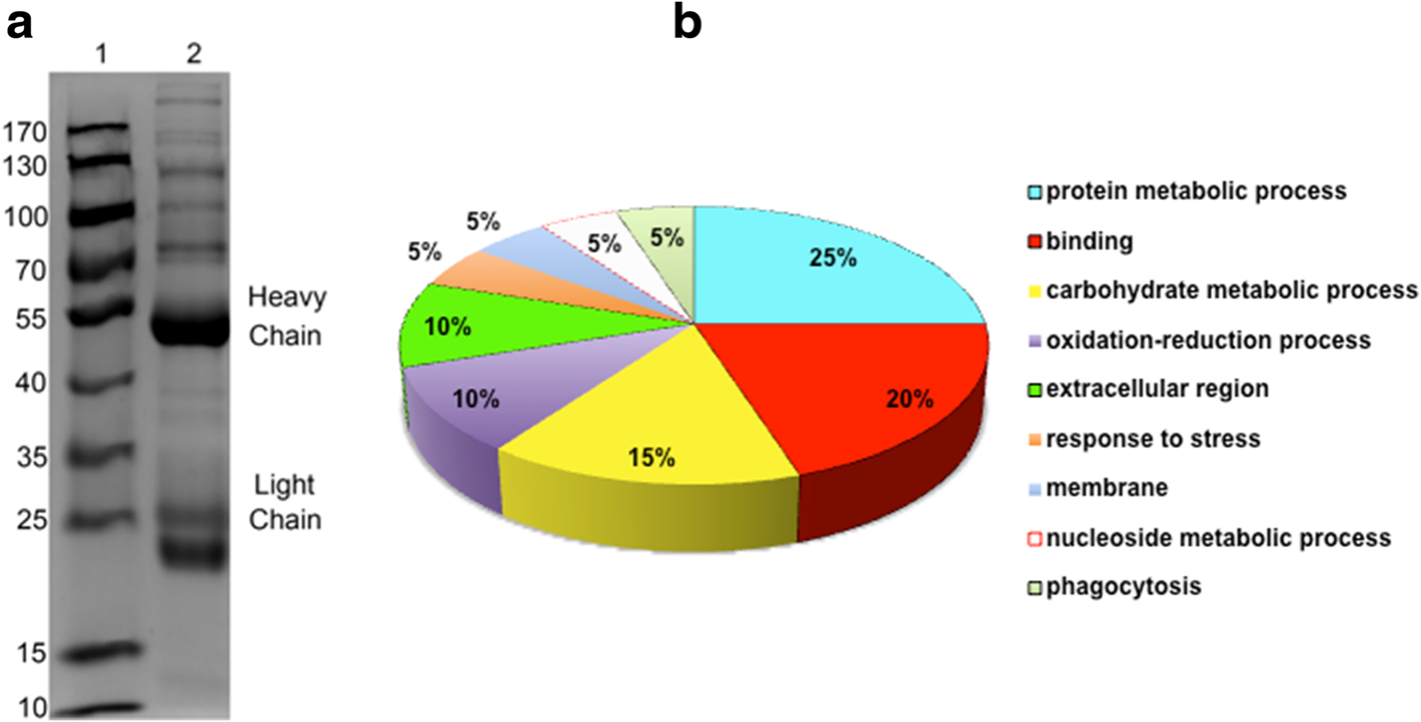
SDS-PAGE analysis and GO annotation of CAgs enriched and purified by immunoprecipitation. **a** Circulating antigens enriched and purified by immunoprecipitation were analysed by SDS-PAGE (8 %). Lane 1: marker; Lane 2: immunoprecipitation supernatant. **b** Pie charts showing the GO distributions for the identified circulating antigens based on the major biological process or cell component categories. Twenty of the identified proteins were successfully annotated

ESA antibodies could be used to identify numerous ESA components with good immunogenicity (Fig. 2). The molecular weights of the vast majority of identified CAgs were greater than 15 kDa (Fig. 3a). In total, 220 protein groups were identified by LC-MS/MS (Additional file 1). Among these proteins, the unique peptide counts for 26 proteins were equal to or greater than 2. The proteins identified by immunoprecipitation-shotgun analysis were classified into the following major categories: micronemal proteins (MIC1, MIC3 and MIC4), dense granule proteins (GRA1 and GRA5), surface antigens (SAG1 and SAG2), novel CAg proteins (coronin, ELMO and ribosomal-ubiquitin protein L40) and others (Table 2).

**Table 2:** CAg proteins identified by LC-MS/MS after IP enrichment and purification with ESA antibodies

Resource ID	Uniprot Database ID	Identified protein name	Peptide count	Unique peptide count^a^	Sequence coverage^b^ (%)	Theoretical Mr (Da)/pI^c^	References of analysis in *T. gondii*	Functional clustering^d^
Micronemes								
1	D8L558	MIC4	4	4	8.62	63,061/5.05	[36, 37]	Protein metabolic process
2	S8F3P1	MIC1	3	3	4.17	48,628/5.15	[37]	Binding
3	T2FG71	MIC3	2	2	7.81	35,165/5.97	[38]	Unknown
Dense granules								
4	P13403	GRA1	4	3	25.79	20,149/4.18	[39]	Extracellular region
5	S7UP40	GRA5	2	2	13.33	12,976/5.78	[40]	Extracellular region
Surface								
6	S7V2Y4	SAG1	4	4	15.77	34,828/8.34	[41]	Membrane
7	I7CQQ5	SAG2	2	2	19.18	14,814/6.17	[41]	Unknown
Novel CAg proteins								
8	S7VXR0	RPL40	3	3	26.36	14,685/9.84	na	Protein metabolic process
9	S8ETG3	Coronin	3	3	3.70	68,441/5.5	na	Protein metabolic process
10	S8GEU1	ELMO	2	2	0.71	243,844/9.91	na	Phagocytosis
11	S8GDY4	Uncharacterized protein	2	2	0.51	295,608/6.23	na	Unknown
12	V4Z4M8	Chorein	2	2	0.11	1,451,666/9.02	na	Unknown
13	V4ZB05	Putative transmembrane protein	2	2	0.99	211,754/4.91	na	Unknown
14	V4ZM84	Uncharacterized protein	2	2	0.65	235,435/5.95	na	Unknown
15	V4YZ80	Ubiquitin-transferase	2	2	0.09	1,711,013/6.68	na	Protein metabolic process
Other								
16	S7VTE3	Protein disulfide isomerase	13	12	29.72	52,801/5.14	[42]	Protein metabolic process
17	P90613	Lactate dehydrogenase	14	11	41.34	35,548/6.03	[43, 44]	Oxidation-reduction process
18	S7VNC9	Actin	9	7	24.39	32,058/5.66	[45]	Binding
19	S8GUS9	ACT1	3	2	4.52	41,907/5.05	[45]	Binding
20	S7W8N1	Chaperonin protein BiP (HSP70)	4	4	5.54	73,252/5.23	[46]	Response to stress
21	S8G950	Enolase 2	3	3	10.53	52,113/6.43	[47]	Carbohydrate metabolic process
22	B9Q0N7	14-3-3 protein	2	2	4.64	37,173/5.05	[48]	Binding
23	S7URH5	Fructose-bisphosphate aldolase	11	11	34.41	46,959/9.01	[49]	Carbohydrate metabolic process
24	S7WD35	Phosphofructokinase PFKII	3	3	3.43	131,361/5.85	[50]	Carbohydrate metabolic process
25	Q2HXR1	Phosphorylase family protein	2	2	8.58	33,042/6.66	[51]	Nucleoside metabolic process
26	S8GL48	Type I fatty acid synthase	2	2	0.21	1,093,457/5.96	[52]	Oxidation-reduction process

^a^Results include proteins that have a unique peptide count ≥ 2

^b^Sequence coverage is defined as the ratio (%) of matched residues in the entire sequence

^c^Theoretical Mr/pI was obtained from Uniprot database

^d^The functions are classified by gene ontology (GO) annotations

*Abbreviation*: *na*, not available

### Bioinformatics Analysis

To better understand the functions of CAgs in biological processes during acute *T. gondii* infection, GO analysis was performed on 26 high-confidence proteins. Twenty proteins were successfully annotated based on biological process or cellular component and classified accordingly (Table 2 and Fig. 3b). Among the annotated proteins, 25 % were involved in protein metabolism, 20 % were involved in binding, 15 % were involved in carbohydrate metabolism, 10 % were involved in oxidation-reduction processes, 10 % localized to extracellular regions, and 5 % were involved in responses to stress, nucleoside metabolism processes and phagocytosis. The other 5 % were membrane components.

### The two CAgs were detected during acute infection by *T. gondii*

**Fig. 4:**
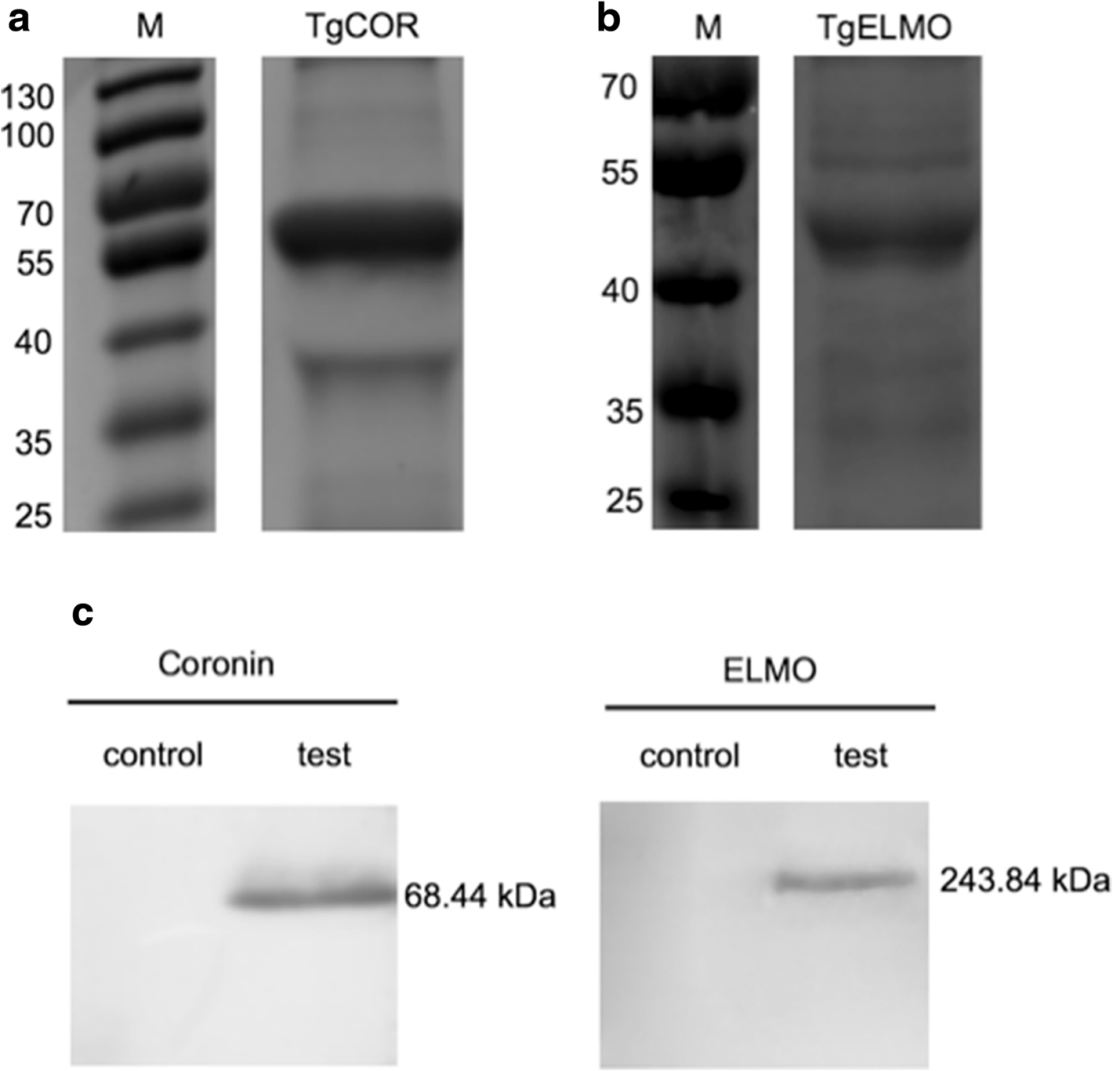
Expression and Western blotting of TgCOR and TgELMO. **a, b** Recombinant TgCOR and TgELMO expressed in *E. coli* were purified with Ni-NTA His-Bind resin. **c** Western blotting of TgCOR and TgELMO. Mixed serum samples from the test group on day 3 were analysed by Western blotting at a dilution of 1:10. Mixed serum samples from the control group were treated as a negative control. The protein bands on the NC membrane were displayed using an ECL kit

**Fig. 5:**
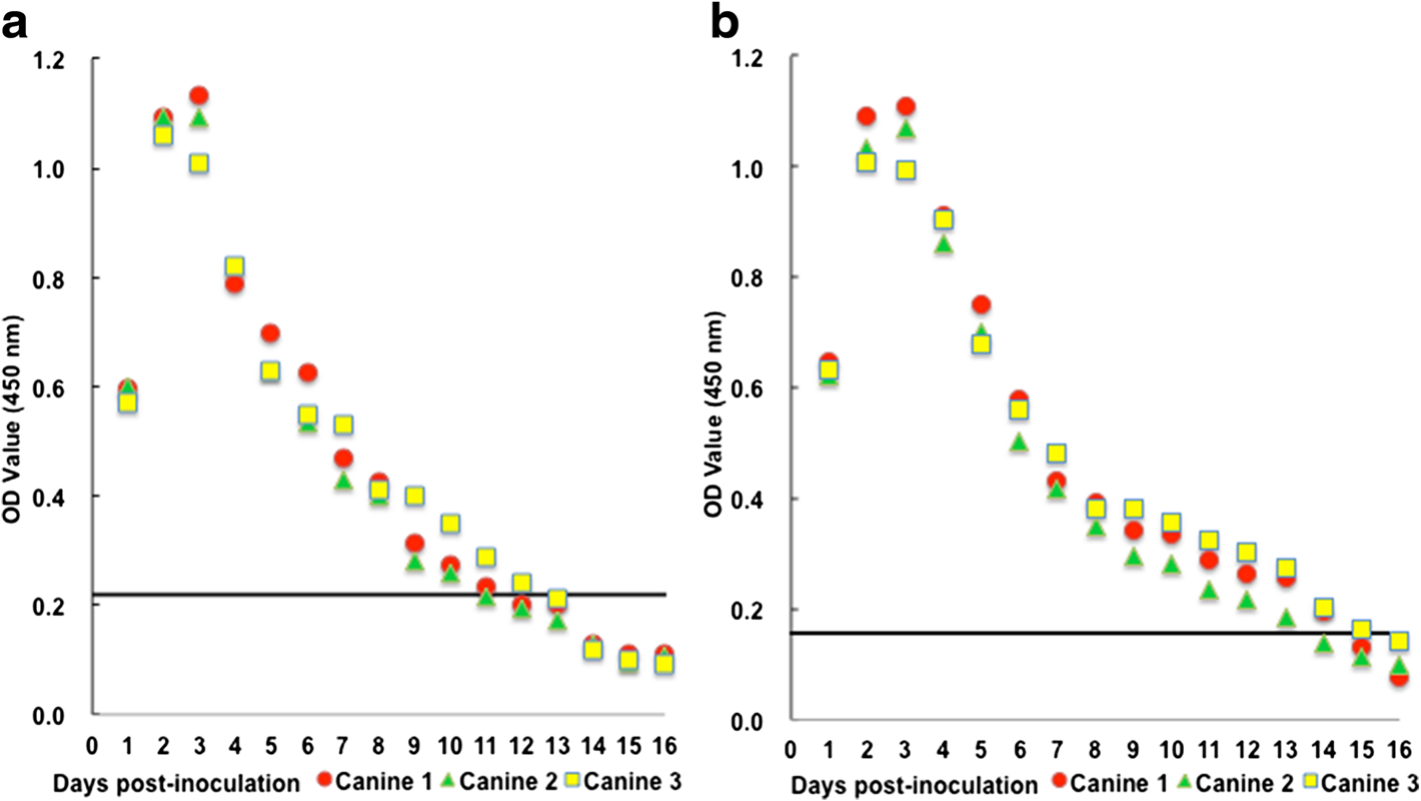
Detection of TgCOR and TgELMO in serum with the ELISA method using antisera. The average OD value for negative samples was multiplied by 2.1 to obtain the cut-off value. The horizontal lines represent cut-off values. The two circulating antigens in the serum of each canine were detected with **a** polyclonal antibodies against recombinant TgCOR and **b** TgELMO

Considering the results of the BLAST analyses of 8 novel CAgs, we chose 2 (coronin and ELMO) with low homology to other species to further assess their diagnostic value. Secondary structure prediction revealed that TgCOR and TgELMO had excellent hydrophilicity and antigenicity in the regions H211–A621 and G1613–L1916, respectively. Peptides containing good hydrophilic domains and antigen epitopes were successfully expressed in an *E. coli* system (Fig. 4a, b). Mixed sera from the test group and control group were analysed by Western blotting. Antiserum for TgCOR or TgELMO was treated as the primary antibody, respectively. The results showed that the 2 novel CAgs identified in this study were present in the sera from canines with acute infections (Fig. 4c). The two circulating antigens reached the highest level between day 2 and day 5. However, TgCOR and TgELMO were undetectable at 12 and 14 days after inoculation, respectively (Figs. 5a, b). TgELMO in the blood was detectable longer than TgCOR, which might be due to the slower clearance rate and lower ELISA detection background of TgELMO.

## Discussion

### Diagnosis of acute *T. gondii* infection

Molecular biological methods (PCR, real-time PCR and Loop-mediated isothermal amplification) can be used to diagnose acute *T. gondii* infection [23, 24]. However, these methods are limited because specific equipment is required. Serological methods have been widely used because they are easy to perform. To date, diagnosis of *T. gondii* infection is mainly based on IgG detection in the blood. However, IgG is only detectable at thirteen days after infection [25]. Also, IgG persists in the blood for a long period and might not represent an acute infection [25]. IgM ELISA and complement fixation tests are the most sensitive methods currently [9], but newborns might not produce IgM antibodies, and IgM production might be delayed because maternal IgG can be transmitted to the foetus through the placenta. Because acute infection might be caused by a recurrent infection, IgM is also rarely produced in immunocompromised patients [10, 26].

As primary toxoplasmosis usually manifests as a flu-like illness in immunocompetent individuals, these cases are often not brought to the attention of the medical personnel and symptoms soon pass without chemotherapy. However, when *T. gondii* infection spreads in humans and animals in an epidemic manner or occurs in immunosuppressed individuals, rapid diagnosis of acute toxoplasmosis becomes particularly important. In cases where acutely infected individuals do seek medical attention, the drugs available for treating influenza do not effectively kill *T. gondii* tachyzoites. Inefficient diagnosis of acute *T. gondii* infection will lead to the spread and treatment delays of toxoplasmosis. We also propose to detect the CAgs of *T. gondii* if the patients experience flu-like symptoms before or during pregnancy. If CAgs tests are positive, emergency strategies such as drug therapies should be taken.

### The presence of circulating antigens in blood represents an acute infection

Serological diagnosis of acute *T. gondii* infection is usually based on the detection of specific antibodies (IgM). However, the presence of IgM antibodies is not always indicative of active toxoplasmosis. IgM can be produced for 6 months after acute infection and remain in the body fluid for up to 18 months or even a few years [27–29]. The presence of IgM during the chronic phase of *T. gondii* infection might lead to unnecessary diagnostic risks and treatment side effects. These risk factors might even lead to the termination of pregnancy [27, 30].

Previous studies have developed acute *T. gondii* infection models in mice or rabbits by intraperitoneal inoculation of tachyzoites. CAgs can be successfully detected with serological methods in animal models [28, 31] and CAgs have also been detected in the blood of rabbits and pigs infected by oral inoculation of oocysts [25]. In the sera of women and children with acute infection, CAgs are also detectable [32]. The previous studies demonstrate that the route of infection does not affect CAgs production. In this study, acute *T. gondii* infection of canines was successfully modelled by intraperitoneal inoculation of tachyzoites. CAgs, markers of acute infection, were also detected in this model.

Acute *T. gondii* infection can be diagnosed by direct detection of CAgs [33]. The diagnosis of acute *T. gondii* infection by CAgs detection is more sensitive than testing for the whole parasite [34]. Although CAgs are partly neutralized by blood antibodies, immunogenic CAgs remain in the blood and can be detected using high levels of specific antibodies [28]. Previous ELISA results showed that the amount of CAgs in women and children with acute infection are significantly higher than that of chronic patients and healthy individuals, which show no differences [32]. CAgs do not persist in the blood for a long period of time [28].

### Selection of multiple CAgs for diagnosis can improve the reliability of diagnostic results

Some CAgs have been used to diagnose acute *T. gondii* infection [31]. SAG1, SAG2, MIC1, MIC3 and GRA1 are proteins identified in this study that have also been studied for use in diagnostic assays [35]. The screening of multiple diagnostic markers is helpful to improve the sensitivity and specificity of diagnosis. The dynamic change of diagnostic candidate molecules in the blood reflects the course of an acute infection. First, the amount of antigen used for serological diagnosis must achieve the minimum detection limit. Multiple molecules represent a large range of CAgs and are more stable than single CAg molecule in the blood. Identification of multiple CAgs and candidate diagnostic molecules will improve the sensitivity of detection for acute infection. Secondly, identification of the protein profile for CAgs will improve the screening range of highly specific antigens and minimize the effect of nonspecific binding on diagnosis. This study identified 26 CAgs components and analysed the preliminary application of two proteins for the detection of CAgs to provide a theoretical basis for the development of sensitive, specific and convenient immunological diagnostic techniques. Furthermore, this study showed that TgCOR and TgELMO are detectable with serological methods during active toxoplasmosis. The two antigens change in a manner consistent with body temperature and *T. gondii* levels in blood and disappear within five days after the disappearance of *T. gondii* in the blood.

However, whether antibodies against two recombinant proteins can be widely used to diagnosis acute infection and whether they can be used to effectively distinguish acute and chronic infections in clinical applications requires a large clinical trial for verification. Diagnosis of *Toxoplasma* by detecting specific CAg also has the potential to reduce the subsequent molecular diagnostic procedures during acute infection. In general, rapid and accurate diagnosis of acute infection will lead to appropriate therapeutic interventions.

## Conclusions

In summary, we identified 26 circulating antigens in the sera of canines infected with *T. gondii*. Among these CAgs molecules, 2 novel candidates were identified as diagnostic antigens for *Toxoplasma*. Based on the described CAgs, diagnostic methods were developed. Further analysis of these methods will be performed in the future and represents improvement in the detection efficiency of *Toxoplasma*.

## Abbreviations

CAg, circulating antigen; DDT, DL-dithiothreitol; DMEM, Dulbecco modified Eagle medium; ECL, enhanced chemiluminescence; ESA, excretory/secretory antigens; GO, gene ontology; GRA, dense granule antigen; HCD, Higher-energy collisional dissociation; IP, immunoprecipitation; LC, liquid chromatography; MIC, micronemal protein; MS/MS, tandem mass spectrometry; NC, nitrocellulose; SAG, surface antigen; SDS-PAGE, sodium dodecylsulfate polyacrylamide gel electrophoresis.

## Additional file

Additional file 1: Table S1. List of CAg proteins identified by LC-MS/MS after IP enrichment and purification with ESA antibodies. (XLSX 27 kb)
